# Design of double functionalized carbon nanotube for amphotericin B and genetic material delivery

**DOI:** 10.1038/s41598-022-25222-1

**Published:** 2022-12-07

**Authors:** Sara Yazdani, Mehrdad Mozaffarian, Gholamreza Pazuki, Naghmeh Hadidi, Idoia Gallego, Gustavo Puras, Jose Luis Pedraz

**Affiliations:** 1 Department of Chemical Engineering, Amirkabir University of Technology, No. 424, Hafez Ave., P.O. Box 15875-4413, Tehran, Iran; 2 Department of Clinical Research and EM Microscope, Pasteur Institute of Iran (PII), Tehran, 1316943551 Iran; 3 NanoBioCel Research Group, Laboratory of Pharmacy and Pharmaceutical Technology, Faculty of Pharmacy, University of the Basque Country (UPV/EHU), Paseo de La Universidad 7, 01006 Vitoria-Gasteiz, Spain; 4 Networking Research Centre of Bioengineering, Biomaterials and Nanomedicine (CIBER-BBN), Institute of Health Carlos III, Av Monforte de Lemos 3-5, 28029 Madrid, Spain; 5 Bioaraba, NanoBioCel Research Group, Calle José Achotegui S/N, 01009 Vitoria-Gasteiz, Spain

**Keywords:** Chemical engineering, Biotechnology

## Abstract

In the present work, single wall carbon nanotubes (SWCNT) were successively functionalized with phospholipid DSPE-PEG carboxylic acid, and then, with ethylenediamine (EDA), to obtain double functionalized single wall carbon nanotube (DFSWCNT). Then, DFSWCNT was applied as a carrier for delivering amphotericin B (Amb) and EGFP plasmid. FSWCNT’s concentration obtained via UV–visible analysis was 0.99 mg/mL. The TGA analysis results provided the lost weights of DSPE-PEG-COOH, EDA, Amb and SWCNT impurities. XPS results showed that carbon atoms’ percentage decreased during the functionalization processes from 97.2% (SWCNT) to 76.4% (FSWCNT) and 69.9% (DFSWNCT). Additionally, the oxygen atoms’ percentage increased from 2.3% (SWCNT) to 21% and 22.5% for FSWCNT and DFSWCNT, respectively. New bonds such as C–N and N–C=O appeared in the synthesized nanocarrier. The I_G_/I_D_ ratio in Raman analysis decreased from 7.15 (SWCNT) to 4.08 (FSWCNT). The amount of Amb released to phosphate buffer saline medium was about 33% at pH = 5.5 and 75% at pH = 7.4 after 48 h. CCK8 results confirmed that the toxicity of functionalized SWCNT had decreased. In a 2:1 ratio of DFSWCNT/EGFP plasmid, the cell viability (87%) and live transfected cells (56%) were at their maximum values. The results indicate that carbon nanotubes have the potential to be applied as drug/gene delivery systems with outstanding properties such as high loading capacity and easy penetration to cell membrane.

## Introduction

Recently, novel drug and gene delivery systems have attracted considerable interest from the research community^1–5^. Nanoparticles^6,7^, hydrogels^8–11^, dendrimers^12,13^, micelles^14,15^ and liposomes^16–18^ are some of the delivery systems that can be modified according to their size, shape, and inherent chemical properties, and used as nanocarriers of pharmaceutical ingredients^19^. Such nanocarriers can increase bioavailability, enhance stability and provide sustained delivery^20^. In addition, biological molecules and drugs can be protected against physical and chemical degradation during their journey to the target location^21^. Furthermore, side effects can decrease considerably, and treatment compliances improve as well. One of the most applicable nanocarriers is carbon nanotubes (CNTs)^21–23^.

CNTs were discovered in 1991 by Ijimia^24^. There are two types of CNTs: single-wall carbon nanotubes (SWCNT) and multi-wall carbon nanotubes (MWCNT), categorized according to the number of their graphene layers^25,26^. SWCNTs are made from one rolled layer of graphene with a 1–2 nm diameter, and MWCNTs are made from two or more rolled layers of graphene with diameters of up to 100 nm^27^. CNTs have become the attractive candidates for different applications in biomedical engineering, biotechnology and pharmaceutical nanotechnology due to their unique optical, thermal, mechanical, electrical and physicochemical properties such as high surface area to volume ratio, their capability to be functionalized and their high capacity of drug/gene loading^25,28–31^. Furthermore, CNTs can deliver hydrophobic and large macromolecules and genetic materials to target sites of action, and deliver two or more therapeutic ingredients simultaneously^32^. Interestingly, the nano-needle shape of CNTs can protect genetic material form enzymatic digestion and enhance permeability of the cell membrane^33^.

Despite such relevant advantages, toxicity and poor solubility are issues that limit clinical applications of CNTs in pharmaceutical industry. To overcome such problems, CNTs can be functionalized with biocompatible molecules^34,35^. Due to the unique architecture and significant CNT’s properties, different industries have utilized them for specific targets. Some of the most important CNT applications are in pharmaceutical industries and medical establishments. Drug vectors, gene delivery to cells or organs, tissue engineering and biosensor diagnostics are some of biomedical CNT applications^36,37^. Benincasa et al. loaded Amphotericin B (Amb) on the surface of functionalized CNT and studied the antifungal activity of Amb against clinical fungal strains, and compared the results with pure and micellar forms of Amb^38^. Masotti et al. functionalized MWCNT with polyethyleneimine and polyamidoamine for delivering miRNA to endothelial cells, and concluded that polymer-coated CNTs had less toxicity than pure CNT^39^. Yan et al. modified MWCNT with polyethyleneimine at the onset, and then with folic acid. Finally, they loaded doxorubicin on the modified MWCNT, and studied the cell viability of doxorubicin and doxorubicin loaded MWCNT in HeLa cells, concluding that doxorubicin loaded MWCNT was nontoxic, and had cell viability values above 90%^40^. Kaboudin and coworkers functionalized MWCNT magentically with pyridine groups and applied them for both plasmid DNA and aptamere formulation deliveries. They demonstrated that binding nucleics to MWCNT can be controlled by an external magnetic field^41^. Cao and coworkers functionalized SWCNT with polyethylenimine and betain, to co-deliver both siRNA (small-interfering RNA) and doxorubicin^42^. Zhao et al. modified the surfaces of SWCNT and MWCNT with peptide lipide and sucrose laurate to obtain a siRNA delivery system, which had high temperature-sensitivity and photothermal performance^43^. Kofoed Andersen et al. applied two kinds of SWCNTs (Hipco- and carboxyl-SWCNT) for methotrexate and a siRNA targeted deliveries^44^. They reported methotrexate efficiencies higher than 70% for both types of SWCNTs. They also reported that the binding efficiency of siRNA was higher than 90% for Hipco-SWCNT and around 80% for carboxyl-SWCNT^44^.

Amb is one of polyene antifungal drugs prescribed for treatment of fungal infections such as leishmaniasis and aspergillosis^45,46^, histoplasmosis^47^, cryptococcal meningitis^48^ and zygomycosis^49^. Its chemical structure includes a polar hydroxylated portion and a nonpolar hydrocarbon sequence. Hypotension, vomiting, headache, and thrombophlebitis are its main adverse effects. Its toxicity and drug side effects are related to its low solubility and narrow therapeutic window.

In this study, we developed a soluble form of CNT to improve Amb’s intrinsic insolubility and also decrease its intrinsic cytotoxicity. Additionally, this new structural form of CNT is a promising candidate for EGFP plasmid delivery, indicating that it can also be used in gene therapy. We used *1, 2-distearoyl-sn-glycero-3-phosphoethanolamine polyethyelen glycol carboxylic acid* (DSPE-PEG-COOH) for SWCNT functionalization to create a biocompatible SWCNT capable of delivering both Amb and EGFP plasmid. In fact, DSPE-PEG-COOH phospholipid has an amphiphilic structure that includes a hydrophobic core (DSPE) and a hydrophilic shell (PEG)^50^, which is widely used for delivering bioactive drugs. It is biocompatible, nontoxic and prolongs circulation time that will enhance absorption of medical agents^50^. Functionalized carbon-nanocarriers were characterized extensively in physicochemical terms, through thermogravimetric analysis (TGA), X-ray photoelectron spectroscopy (XPS), size distribution, zeta potential, and Raman spectroscopy. In addition, surface morphology was evaluated by both scanning and transmission electron microscopy techniques (SEM and TEM). Also, the release of Amb from loaded CNTs into two PBS mediums with different pH values (5.5 and 7.4) was investigated, and the kinetic release profile was determined by data analysis. Similarly, SWCNT’s cytotoxicity before and after functionalization was evaluated through a CCK8 assay. Moreover, cytotoxicity analyses of pure Amb and Amb loaded SWCNT were performed, and the results were compared. In addition, EGFP plasmid was added to DFSWCNT at different DFSWCNT/EGFP plasmid ratios (w/w) to study the transfection efficiency in HEK-293 cell, in terms of the percentages of transfected and live cells.

## Materials and methods

Pure SWCNTs and DSPE-PEG (5000)-COOH were purchased from NanoCs Company (USA) and NOF-Sunbright Company (USA), respectively. Amphotericin B (Amb), ethylenediamine (EDA) and Poly-L-lysin hydrobromide were purchased from Sigma-aldrich, (USA). 1-ethyl-3[3-dimethylaminopropyl] carbodiimide hydrochloride (EDC), N-hydroxysulfosuccinimide (sulfo-NHS), 2-(N-morpholino) ethanesulfonic acid (MES) buffer saline, and phosphate buffer saline (PBS) were all purchased from Thermo Scientific Company (USA). Escherichia coli DH5α was used to propagate the reported CMS-EGFP plasmid (5.5 kb, PlasmidFactory, Germany), named pEGFP. Then, according to manufacturer´s instructions, the pEGFP was purified using the Qiagen endotoxin-free plasmid purification Maxi-prep kit (Qiagen, Santa Clarita, CA, USA). The final pEGFP’s concentration was quantified by measuring the absorbance by a SimpliNano™ device (GE Healthcare, Buckinghamshire, UK) at 260 nm. Cell counting kit-8 (CCK8; Sigma-Aldrich, Madrid, Spain), 0.25% trypsin–EDTA (Gibco, San Diego, CA, USA), serum-free Opti-MEM solution (Gibco, California, USA), lipofectamine™ 2000 transfection reagent (Invitrogen, Carlsbad,CA,USA) and propidium iodide (Sigma-Aldrich, Madrid, Spain) were procured. HEK-293 (ATCC CRL-1573) cell lines were purchased from ATCC (manassas VA, USA). Cells were grown in Eagle’s Minimum Essential Medium (EMEM, 30–2003, ATCC-Manassas, VA, USA) supplemented with 10% of fetal bovine serum (FBS) (Sigma-Aldrich, Madrid, Spain) and 1% of antibiotic/antimycotic solution (A/A) (Life Technologies, Paisley, UK). Milli-Q water was used in all experiments.

### Preparation of CNT and conjugation to Amb and EGFP plasmid

#### Functionalizing SWCNT with DSPE-PEG (5000)-COOH

**Figure 1:**
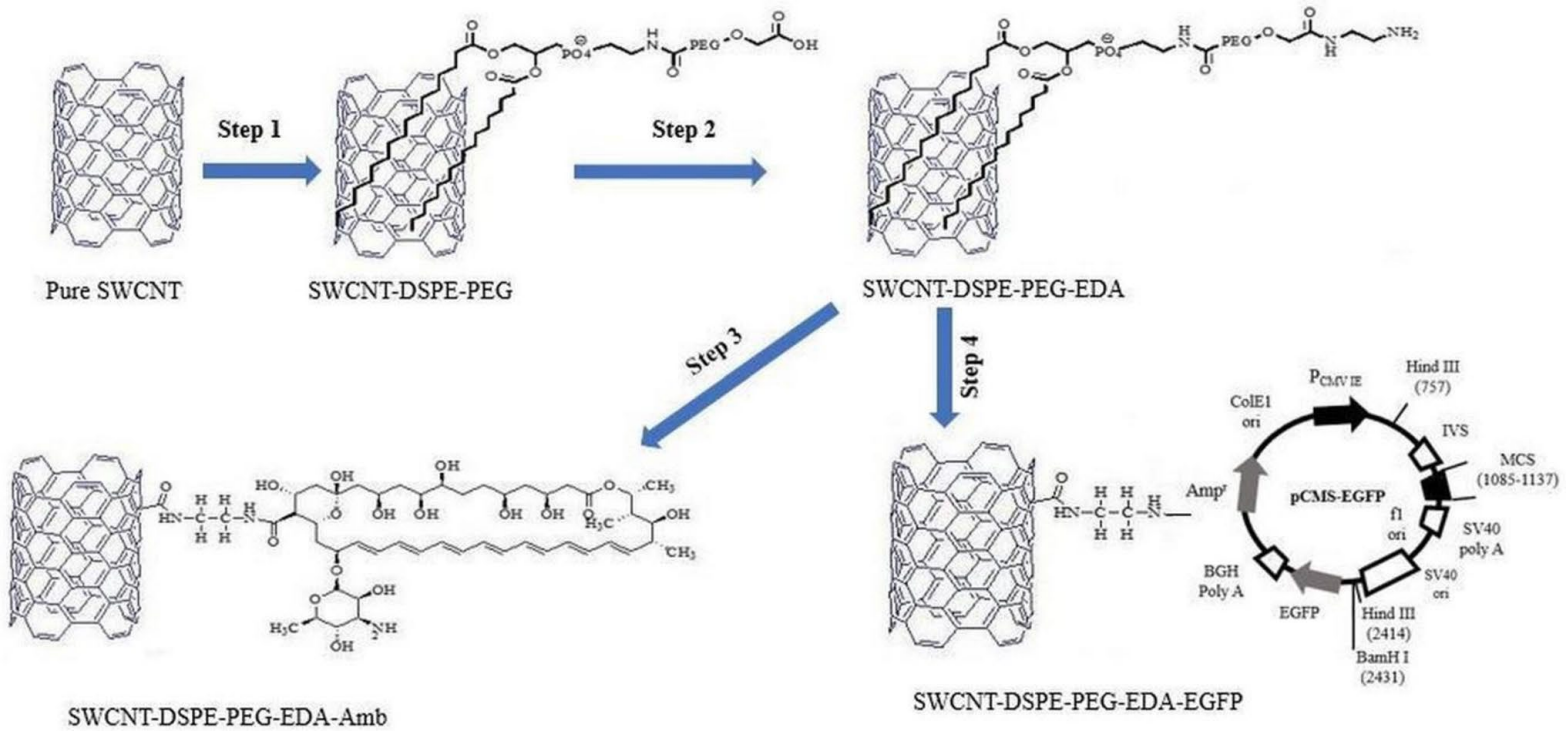
Schematic display of attaching DSPE-PEG to SWCNT (called FSWCNT, step 1), and conjugaing EDA to FSWCNT (called DFSWCNT, step 2), Amb (step 3) and EGFP (step 4) to DFSWCNT.

In the first step, based on the modification of the procedure described by Hadidi and coworkers^51^, pure SWCNT was functionalized non-covalently with DSPE-PEG(5000)-COOH with a SWCNT/DSPE-PEG-COOH weight ratio of 0.2, and the product was called functionalized single wall carbon nanotube (FSWCNT). In other words, the FSWCNT was made from dissolving 10 mg SWCNT in 5 mL Milli-Q water and 50 mg DSPE-PEG (5000)-COOH in 5 mL Milli-Q water. Pure SWCNT was dispersed completely through prob-sonicator (about 20 min). The dispersion process was performed in an ice-bath sonicator for 60 min due to heat sensitivity of phospholipid DSPE-PEG (5000)-COOH. In the next step, the dispersed SWCNT and DSPE-PEG (5000)-COOH solutions were mixed together, and sonicated in an ice-bath sonicator for 5 h. The resulting product of this step was centrifuged at 5000 rpm for 30 min at 5 °C. The supernatant portion of this product is the FSWCNT (Fig. 1, step 1).

#### Conjugation of EDA to FSWCNT

In this step, 1.6 mg of EDC was added to 10 mL of FSWCNT with a concentration of 1 mg/mL. EDC is a carboxyl and amine-reactive zero-length cross-linker. The conjugation of carboxylic acid group of FSWCNT to amine groups of EDA is helped by formation of amid bonds between them. Then, 5 mL of EDA was added to the above solution, and sonicated in an ice-bath sonicator for 5 h. Next, this mixture was centrifuged at 5000 rpm for 30 min, and finally dried through lyophilization. The product of this step is called double functionalized single wall carbon nanotube (DFSWCNT), due to being functionalized originally with DSPE-PEG-COOH, and then functionalized again with EDA (Fig. 1, step 2).

#### Attachment of Amb to DFSWCNT

In the final step, based on the procedure by Vosoughi and coworkers^52^, the Amb drug was attached to DFSWCNT as a nanovector. 2 ml of Amb (250 µg/mL) was dispersed in 0.2 mL MES buffer (0.5 M), and 0.46 mL of NHS aqueous solution (50 mg/mL) was added, and mixed completely by a stirrer. Then, 0.24 mL EDAC solution (10 mg/mL) was added to the above suspension and stirred at 350 rpmfor 40 min at room temperature. On the other hand, DFSWCNT was re-dispersed in 9 mL of MES buffer solution (0.05 M) with $\mathrm{pH}\sim$ 6.1, and added to the Amb suspension. The final product was mixed on a shaker (350 rpm, 60 min) and centrifuged (5000 rpm, 20 min). Finally, the product was washed three times with 0.05 M MES buffer to remove excess Amb. The product of this step is DFSWCNT-Amb (Fig. 1, step 3).

#### Conjugation of EGFP plasmid to DFSWCNT

Complexes were obtained by adding an appropriate volume of EGFP plasmid stock solution to DFSWCNT solution based on 6 different DFSWCNT/EGFP plasmid mass ratios (w/w). These complexes were incubated for 30 min at room temperature before application, to promote the electrostaic interaction between the amine groups of DFSWCNT and phosphate groups of EGFP plasmid DNA, and obtain the desired complexes (Fig. 1, step 4).

### Physicochemical characterization of nanocarrier

#### UV–visible assay

UV–visible spectrophotometry (Infinite M200, TECAN, Spain) was applied to determine: FSWCNT’s concentration (at 256 nm wavelength), the percentage of Amb loading on DFSWCNT system and draw the calibration curves of Amb in Milli-Q water/PBS at pH values 5.5 and 7.4. The calibration curve of P7-SWCNT as standard CNT, which is described with the adjacent Eq. (1)^51^, was used for determination of FSWCNT’s concentration. In order to draw Amb’s calibration curve, different concentrations of pure Amb (in Milli-Q water, PBS with pH values 5.5 and 7.4) were made, and their absorbance values were read at 410 nm wavelength. Finally, the absorbance values were plotted versus concentrations, and linear curves were obtained by linear regression analysis of the corresponding plot points, and used for relevant calculations. All experiments included two repitations.

1 $$ {\text{Absorbance}} = 13.07 \times {\text{Concentration}} \,({\text{mg}}/{\text{mL}}) - 0.0452 $$

#### Thermogravimetric analysis (TGA)

To obtain the mass contents of impurities in SWCNT, DSPE-PEG-COOH, EDA and Amb; TGA analyses of pure SWCNT, FSWCNT, DFSWCNT and DFSWCNT-Amb were performed by TGA/DSC3 + (Mettler Toledo) equipment under N_2_ atmospheric condition in an alumina cell with a flow rate of 30 mL/min and a temperature rate of 10 °C/min. The molar functionality of carbon atoms can be determined by Eq. (2)^53^. On the other hand, the fraction of functionalized carbon atoms can be estimated by determination of DSPE-PEG-COOH loading on pure SWCNT. The molar functionality result of carbon atoms is given in the section for TGA results.

2 $$ {\text{Molar}}\,{\text{functionality}} = \frac{{{\text{total}}\,{\text{mass}}\,{\text{loss}} \times 12}}{{{\text{Mw}}\,{\text{of}}\,{\text{DSPE}} - {\text{PEG}} - {\text{COOH}} \times {\text{mass}}\,{\text{loss}}\,{\text{related}}\,{\text{to}}\,{\text{DSPE}} - {\text{PEG}} - {\text{COOH}}}} \times 100 $$

#### X-ray photoelectron spectroscopy (XPS) assay

Surface characteristics (such as chemical functional groups, chemical bonds and percentage of atomic concentrations) of pure SWCNT, FSWCNT, DFSWCNT and DFSWCNT-Amb structures can be obtained by XPS analysis. In this study, the XPS measurements were obtained by a SPECS system (Berlin, Germany) equipped with a Phoibos 150 1D-DLD analyzer and monochromatic radiation source Al Kα (1486.7 eV). An initial analysis was carried out to determine the elements in the sample (wide scan: step energy 1 eV, dwell time 0.1 s, pass energy 80 eV) and the detailed analysis of the detected elements was carried out (detail scan: step energy 0.08 eV, dwell time 0.1 s, pass energy 30 eV) at an electron take-off angle of 90°. The spectrometer had been previously calibrated with Ag (Ag 3d5/2, 368.26 eV). The spectra were adjusted using the CasaXPS 2.3.16 software, which models the Gauss-Lorentzian contributions after background subtraction (Shirley). The concentrations were calculated by correcting the values with relative atomic sensitivity factors (Scofield).

#### Size, zeta potential and polydispersity determination

The hydrodynamic diameter, which includes particle size, reported as mean particle intensity, and the polydispersity index (PDI) of nanocarriers were measured by Dynamic Light Scattering (DLS), and the Zeta potential was measured by Lasser Doppler Velocimetry (LDV) in a Zetasizer Nano ZS (Malvern Instrument, UK)^54^. To carry out the measurements, 50 µL of each sample was diluted in 950 µL of 0.1 mM NaCl solution. The particle’s hydrodynamic diameter was obtained by cumulative analysis. The Smoluchowski approximation supported the calculation of Zeta potential based on electrophoretic mobility. All measurements were repeated three times.

#### Raman spectroscopy assay

The measurements were carried out by a Renishaw InVia Raman spectrometer, attached to a Leica DMLM microscope. The spectra were acquired with a Leica 50 × N Plan (0.75 aperture) objective. Additionally, to visualize and focus, another Leica 5 × N Plan (0.12 aperture) and a 20 × N Plan EPI (0.40 aperture) object were also used. The spatial resolution for the 50 × objectives is 2 microns. The microscope is equipped with a Prior scientific motorized stage (XYZ) with a joystick to facilitate focusing on or searching the points of interest. The excitation wavelength was 514 nm (ion-argon laser, Modu-Laser) with 50 mW of nominal power at the source, and 20 mW of maximum power directed to the sample. A holographic net of 1800 lines/mm was employed. The power of the laser was reduced by neutral density filter in all of the measurements to avoid photo-decomposition of samples (burning). Each spectrum took 20 s, and 5 scans were accumulated with 10% of the maximum power of the 514 nm laser in the 150–3500 cm^−1^ spectral window.

#### Morphological studies (SEM, TEM)

Morphologies of pure SWCNT, FSWCNT, DFSWCNT and DFSWCNT-Amb were assessed by transmission and scanning electron microscopy analyses (TEM, SEM). Solid pure SWCNT was suspended in ethanol, sonicated, and one of its droplets was deposited on a Cu TEM grid with Holley carbon film. Likewise, one droplet from each of the other samples was also deposited on individual Cu TEM grids with Holley carbon film. TEM bright field images were obtained with a Philips (Eindhoven, The Netherlands) CM200 TEM, operating at an accelerating voltage of 200 kV. For SEM analysis, samples of FSWCNT, DFSWCNT and DFSWCNT-Amb were diluted in Milli-Q water at a 1:10 ratio, and deposited on an Au coated glass cover. Secondary electron images were obtained with a JEOL (Tokyo, Japan) JSM-7000-F SEM, equipped with a Schottky field emission gun. The microscope was operated at 10 kV, and an applied probe current of about 0.01 nA.

### Amb release from DFSWCNT

As time goes on, Amb particles will detach from nanocarrier and diffuse to the release medium. The amount of Amb in release medium was quantified by measuring Amb’s absorbance in release medium by UV–visible analysis at 410 nm wavelength. Amb’s release percentage and its releasing kinetic can be determined with the help of a calibration curve. So, 2 mL of DFSWCNT-Amb formulation was poured into a dialysis bag and put in a 20 ml PBS solution at two pH settings (5.5 and 7.4), and placed on a shaker operating at 100 rpm for a period of 48 h at 37** °C**. 3 mL of the PBS solution was withdrawn at specific time intervals during a 48 h time span, and analyzed directly through a UV–visible spectrophotometer. In order to keep the sink conditions constant, 3 mL of fresh PBS was added frequently to the release medium at specific points of time during all of the in vitro release studies. In order to investigate the effect of pH on Amb releasing process, two release PBS mediums with different pHs were set up and studied. All experiments were performed three times.

### Cell culture

HEK-293 cells were maintained as a monolayer culture in EMEM medium with Earle’s Balanced Salt Solution (BSS) and 2 mM l-glutamine supplemented with 10% heat-inactivated FBS and 1% A/A. The cells were incubated at 37° C under 5% CO_2_ atmosphere and sub-cultured every 2–3 days.

### Viability assay

The cells were seeded using EMEM media without A/A, at a density of 15,000 cells per well in 96 well plates, and allowed to adhere overnight to reach 70–80% confluence for the biocompatibility assay. The CNT stock solution was diluted in EMEM media without A/A to achieve a concentration of 1 mg/mL, and sonicated for 30 min. At those conditions, cells were exposed to pure SWCNT, FSWCNT and DFSWCNT at concentrations ranging from 0.075 to 1 mg/mL for a period of 24 h at 37 °C under appropriate controls. Then, 10 µL/well of CCK8 reagent was added to each well and incubated for 4 h, and the absorbance was read with a microplate reader at 450 nm (Infinite M200, TECAN, Spain). Furthermore, in order to compare the cytotoxicity of Amb and Amb loaded on the surface of DFSWCNT, appropriate amounts of both Amb and (DFSWCNT-Amb) solutions were lyophilized to obtain different concentrations of Amb and DFSWCNT-Amb compounds in EMEM media without A/A. To achieve this, HEK-293 cells were exposed to pure Amb and DFSWCNT-Amb at concentrations ranging from 1.431 to 37.5 µg/mL for a period of 24 h at 37 °C under appropriate controlled conditions. Finally, the cytotoxic analyses of pure Amb and DFSWCNT-Amb were performed by CCK8 assay as described before. At least four wells were allocated for each cocentration.

### Gene delivery efficiency

To evaluate the transfection efficiency, HEK-293 cells were seeded using EMEM media without A/A into 24 well plates previously pre-treated with poly-lisin, at an initial density of 20 × 10^4^ cells per well, to reach 70–80% of conflunece at the time of transfection assay. Next, the cells were exposed to DFSWCNT-EGFP complexes at different mass ratios in OptiMEM medium and incubated for 4 h at 37 °C (5% CO_2_). Then, the transfection medium was discarded and the cells were washed with PBS. Afterwards, 500 µL of fresh EMEM medium containing 10% FBS per well was added and the cells were allowed to grow for 48 h. Commercially available Lipofectamine™ 2000 transfection reagent was used as a positive control^55^.

FACSCalibur flow cytometry (Becton Dickinson Bioscience, San Jose, USA) was used to perform flow cytometry analysis to quantify the percentage of live cells, after satining with propidium iodide (FL3 channel, 650 nm), and transfected with DFSWCNT-EGFP plasmid complexes (FL1 channel, 525 nm). For this purpose, at the end of the incubation time, the cells were washed with PBS and detached from the 24 wells with 0.25% trypsin/EDTA (200 µL per well). Then, trypsin activity was neutralized with supplemented EMEM growth medium without A/A and the cells were prepared and analyzed by flow cytometry as described before^55,56^.

## Results and discussion

### UV–visible

The absorbance value of FSWCNT’s diluted sample was read by UV–visible spectrophotometry at 256 nm wavelength. This absorbance value was substituted in P7-SWCNT’s calibration curve equation^51^, and its calculated concentration was 0.99 mg/mL. Also, the absorption spectrum of Amb standard aqueous solution (250 µg/mL) indicated strong absorption at 410 nm wavelength, which was in line with other researches^57,58^. Therefore, the completely purified DFSWCNT-Amb’s absorbance was read through UV–visible analysis at 410 nm, and then by using the calibration curve of Amb in Milli-Q water equation shown in supplementary Figure S1, the achieved loading percentage of Amb on DFSWCNT was determined to be 54%.

### TGA

**Figure 2:**
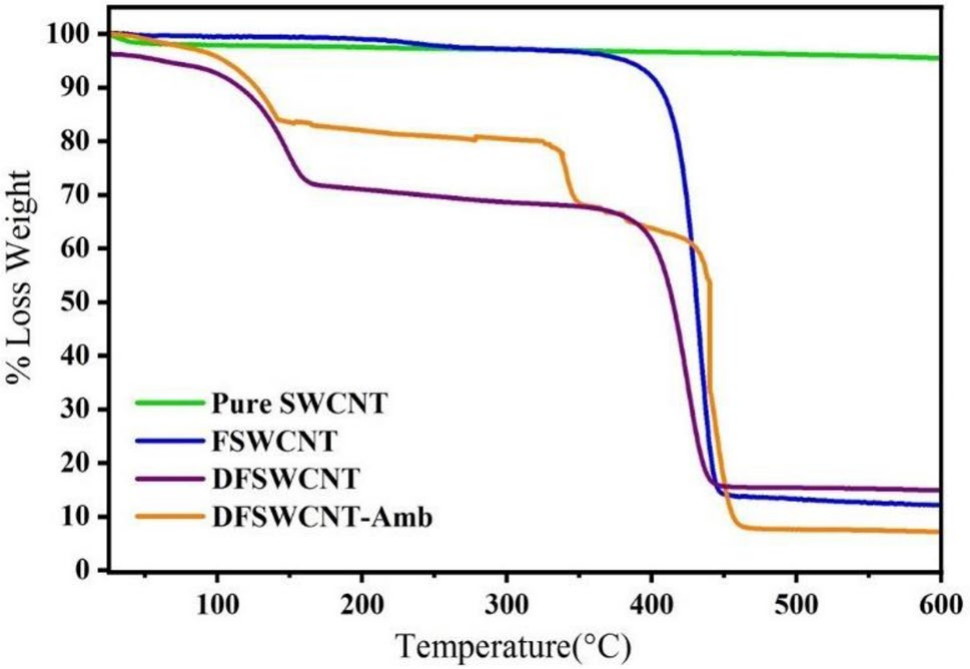
Thermogravimetric diagram for SWCNT, FSWCNT, DFSWCNT and DFSWCNT-Amb.

Figure 2 displays TGA analysis results. It can be seen that the mass losses related to SWCNT, DSPE-PEG-COOH, EDA and Amb occurred at ranges (540–800) °C, (180–540) °C, (25–180) °C and (300–400) °C, respectively. Comparing TGA results of FSWCNT with pure SWCNT indicate that the total FSWCNT sample’s mass loss was about 93%, consisting of 86% loss due to DSPE-PEG-COOH and 7% related to SWCNT impurities. Total mass loss in DFSWCNT was near 95% consisting of 21.5%, 66.5% and 7% related to EDA, DSPE-PEG-COOH and SWCNT impurities, respectively. Total mass loss in DFSWCNT-Amb was almost 97% including 20%, 58% and 7% related to EDA, DSPE-PEG-COOH and SWCNT impurities, respectively. Also, Amb’s mass loss in DFSWCNT-Amb sample was 12%, which was nearly consistent with the UV analysis result. The total mass loss, molecular weight of DSPE-PEG-COOH and mass loss related to DSPE-PEG-COOH in Eq. 2 were 93%, 5898 and 86%, respectively. Therefore, the molar functionality obtained for FSWCNT was 0.22. This value indicates that 0.22% of carbon atoms in pure SWCNT were functionalized during the process. The weight loss of pure SWCNT was considered as a base reference in all of the above TGA calculations. Also, the detailed TGA values for pure SWCNT, FSWCNT, DFSWCNT and DFSWCNT-Amb are reported in Table S1.

### XPS

**Figure 3:**
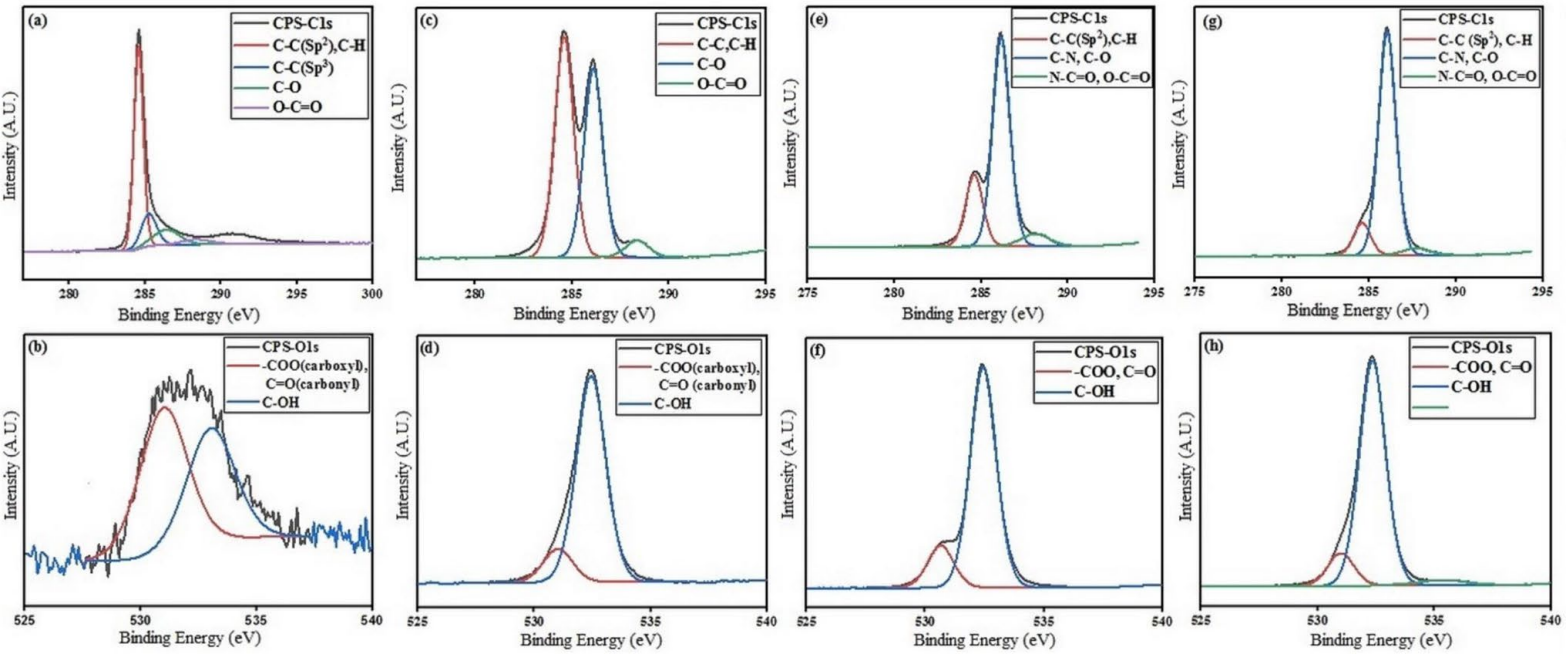
X-ray photoelectron spectroscopy (XPS) C1s and O1s high resolution spectra (**a**) C1s pure SWCNT, (**b**) O1s pure SWCNT, (**c**) C1s FSWCNT, (**d**) O1s FSWCNT, (**e**) C1s DFSWCNT, (**f**) O1s DFSWCNT, (**g**) C1s DFSWCNT-Amb and (**h**) O1s DFSWCNT-Amb.

Figure 3 shows the C1s and O1s high resolution spectra for pure SWCNT, FSWCNT, DFSWCNT and DFSWCNT-Amb. Supplementary Tables S2, S3, S4 and S5 represent C1s, O1s, N1s and P2p, respectively related results, including functional groups, binding energy (BE) and percentage of SWCNT atomic concentrations at each step^59^. The C1s XPS spectrum for pure SWCNT in Fig. 3a shows four peaks at 284.6, 285.2, 286.4 and 288.4, which were assigned to C–C(sp^2^) or C–H, C–C(sp^3^), C–O and O–C=O, respectively^59,60^. As the results have shown, after functionalizing SWCNT with DSPE-PEG-COOH, the contribution related to C-O group increased from 11.4% (pure SWCNT) to 33.6% for FSWCNT, and the overall percentage of carbon decreased from 97.2% (pure SWCNT) to 76.4% (FSWCNT). As FSWCNT functionalized with EDA, and EDA’s amine group attached to the carboxylic acid group in phospholipide DSPE-PEG-COOH, then functional groups such as C–N and N–C=O appeared in DFSWCNT’s C1s spectrum. As for DFSWCNT, the contribution related to C–N and N–C=O groups increased to 48.6 and 4.5, respectively. This confirmed the attachment of EDA to FSWCNT. On the other hand, as Amb loaded on DFSWSCNT, the high number of C-O bonds in Amb structure was being revealed in DFSWCNT-Amb’s C1s spectrum. Also, the results of O1s high resolution spectra for SWCNT before and after functionalization with DSPE-PEG-COOH showed that the percentage of oxygen atom increased considerably after functionalization. The percentage of –COO (carboxyl group) or C=O (carbonyl group) increased from 1.3% (pure SWCNT) to 2.9% (FSWCNT). The concentration percentage of C–OH group increased considerably from 1.0% (pure SWCNT) to 18.1% (FSWCNT). Overall, the percentage of oxygen atoms in FSWCNT reached to 21%. However, the percentage of oxygen atoms in DFSWCNT did not change considerably due to lack of oxygen atoms in EDA. However, in the DFSWCNT-Amb formulation, the high number of C-O bonds in Amb structure affected the percentage of this bond, and it increased from 18.5 (DFSWCNT) to 24.3(DFSWCNT-Amb). Figure S2 shows the N1s and P2p high resolution spectra for FSWCNT, DFSWCNT and DFSWCNT-Amb. Table S4 includes information related to presence of nitrogen atoms in FSWCNT, DFSWCNT and DFSWCNT-Amb structures. Nitrogen and phosphorus atoms were not observed in pure SWCNT. Results show that the concentration of nitrogen atoms inceased considerably from 1.11% (FSWCNT) to 5.21% (DFSWCNT), and that is due to nitrogen atoms existing in EDA’s structure, which confirms the conjugaton of EDA to FSWCNT. The concentration of nitrogen atoms in DFSWCNT (5.21%) is close to that of nitrogen atoms in DFSWCNT-Amb (5.50%), which is due to the existence of nitrogen atoms in the Amb structure. On the other hand, due to the existance of phosphorus atoms in DSPE-PEG-COOH structure, phosphorus atoms appear in the P2p high resolution spectra for FSWCNT, DFSWCNT and DFSWCNT-Amb. Table S5 includes information relating to existance of phosphorus atoms in FSWCNT, DFSWCNT and DFSWCNT-Amb structures. Results indicate that the concentrations of phosphorus atoms in the three samples (FSWCNT, DFSWCNT, DFSWCNT-Amb) are similar to each other and are about 0.32%. Finally, in this study, the effects of conjugating ingredients to SWCNT (such as surface chemistry and atoms quantity) were visible in XPS analysis results, and this was a way to confirm the modification of SWCNT just like in other researches^61,62^.

### Size, polydispersity and zeta potential

**Figure 4:**
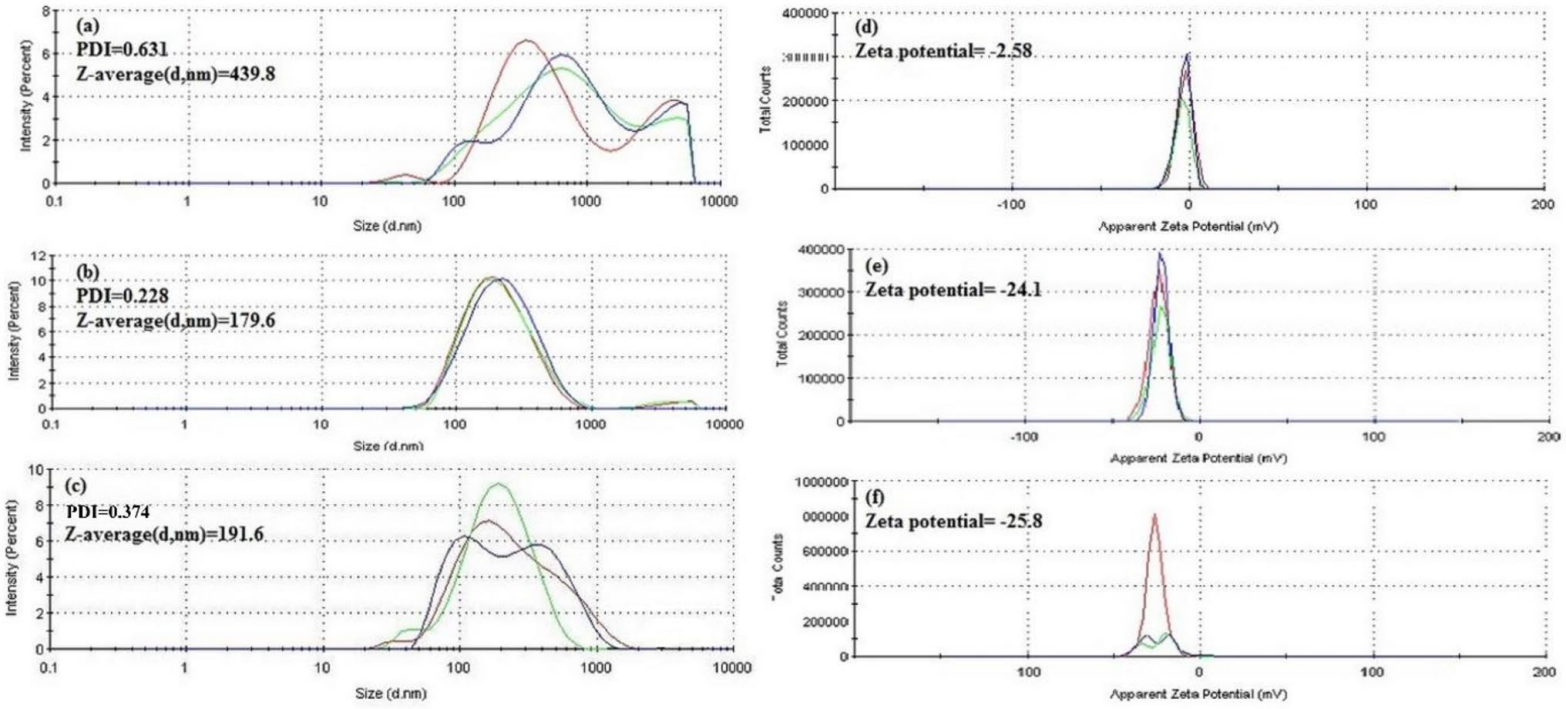
Size distribution and polydispersity index (**a**) Pure SWCNT, (**b**) FSWCNT and (**c**) DFSWCNT. Zeta potential of (**d**) Pure SWCNT, (**e**) FSWCNT and (**f**) DFSWCNT.

As can be seen in Fig. 4, SWCNT’s functionalization improved relevant physicochemical parameters such as size, polydispersity and zeta potential that would impact their final biological performance. The DLS’s size-distribution profiles, showed a high curve shifted to the right in the micrometer scale for SWCNT with a mean size of 439.8 nm and a PDI value of 0.631 (Fig. 4a). In contrast, DFSWCNT presented a nanometer main curve with a 191.6 nm mean size and a PDI value of 0.374 (Fig. 4c). Hence, both size and PDI values were reduced after functuionlization, which would result in a smaller and more homogenueous dispersion. Although DLS may not be the most accurate method for the physicochemical characterization of elongated structures such as carbon nanotubes, this strategy has been widely used in the literature for this purpose^63–66^.Interestingly, the PDI data obtained with our DSWFCNT are similar to those reported in the previously cited literature.

Regarding the zeta potential value, which is a widely used parameter to evaluate dispersion stability, our data showed that DFSWCNT had a more negative zeta potential value (-25.8 mV, Fig. 4f) compared with SWCNT (-2.58 mV, Fig. 4d). These data reveal that functionalizing SWCNT enhances dispersion stability due to electrostatic repulsive forces between negatively charged particles, that would avoid agregates, in accordance with previously published literature^67–69^. In fact, the dispersion stability of DFSWCN was maintained over a period of 4 months at 4 °C, without visual sign of any aggregate formation (supplementary Figure S3).

### Raman spectroscopy

**Figure 5:**
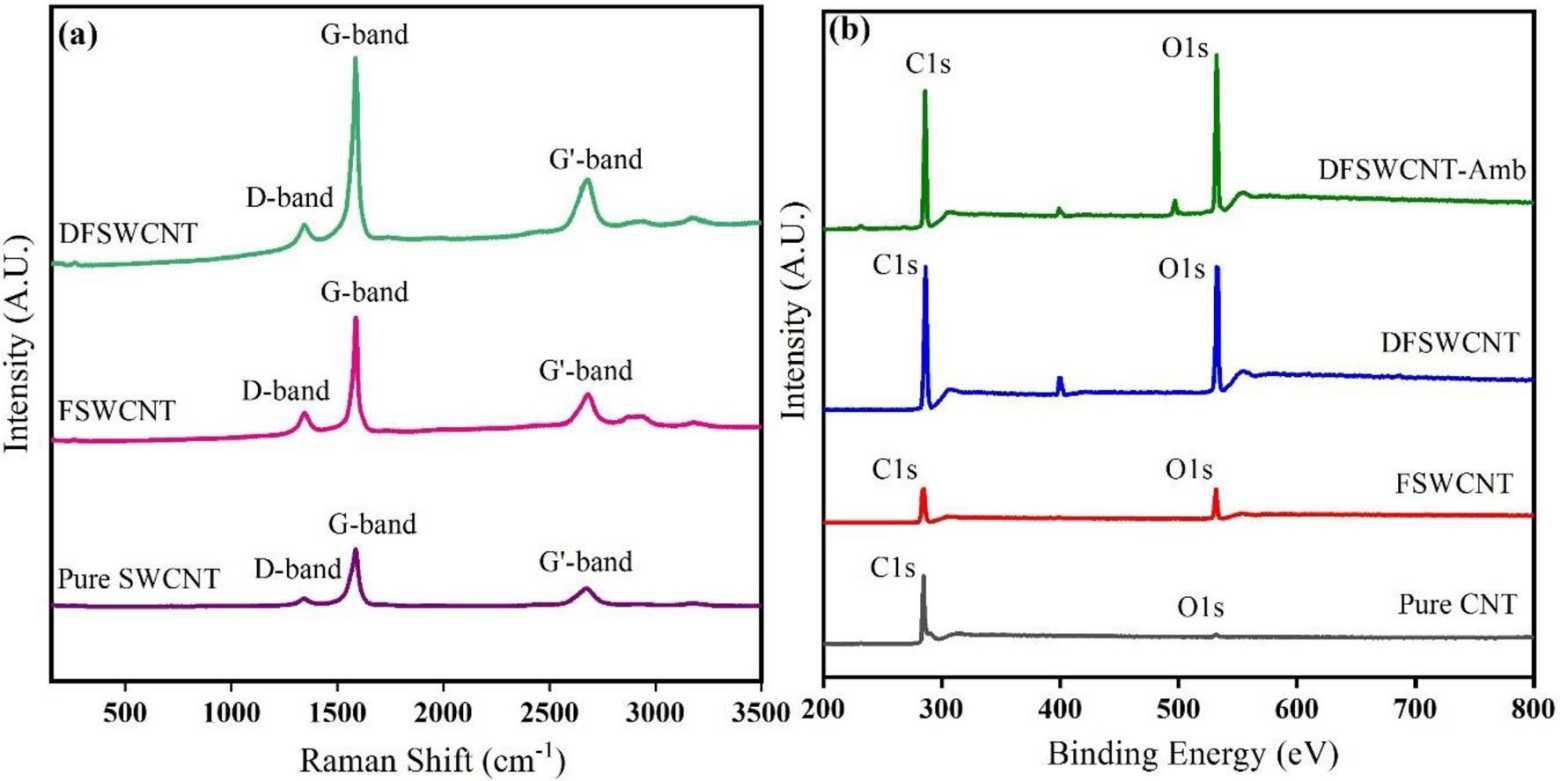
(**a**) Raman Analysis and (**b**) wide-scan C1s and O1s spectra for SWCNT, FSWCNT, DFSWCNT and DFSWCNT-Amb.

Raman Spectroscopy is a powerful technique for investigation of the structural variations and properties of carbon nanotube before and after modification^62^. The attachments of DSPE-PEG-COOH to SWCNT and EDA to FSWCNT could be investigated quantitatively based on the intensity of G-band to D-band ratio (I_G_/I_D_) and the Raman shift of D or G-bands. The D-band represents the defective or disordered sites on CNT as well as the presence of impurities that mostly occurred at 1330 ± 20 cm^−1^^70,71^. The G-band is related to the tangential vibrating mode of carbon atoms and it usually appeared at 1550–1600 cm^−1^^71,72^. The Raman spectra of SWCNT, FSWCNT and DFSWCNT are shown in Fig. 5a, with the results of Raman analysis displayed in Table 1. In the Raman spectrum, the pure SWCNT’s specified peak of D-band was at 1341 cm^−1^ and the peak of G-band was at 1585 cm^−1^. Also, a clear peak at 2674 cm^−1^ was related to G'-band, which is related to the second ordered process or radial movement of carbon atoms, and appeared to be close to twice the size of D band. In FSWCNT, the peaks of D-band , G-band and G'-band shifted to 1347 cm^−1^, 1588 cm^−1^ and 2682 cm^−1^, respectively. The intensity of G-band to D-band was an important criterion for ensuring that carbon nanotubes were functionalized. The ratio I_G_/I_D_ decreased from 7.15 (pure SWCNT) to 4.08 (FSWCNT). If ratio I_G_/I_D_ had reduced more, it would have been an indication that SWCNT had functionalized better and created more defects on its surface. As DFSWCNT’s Raman results show, I_G_/I_D_ ratio decreased from 4.08 to 3.91, which indicates new defects had been created on FSWCNT’s surface. These defects are related to EDA, which conjugated to FSWCNT. Supplementary Table S6 and Fig. 5b show the wide-scan and wide-scan spectra results of XPS recorded for pure SWCNT, FSWCNT, DFSWCNT and DFSWCNT-Amb. As can be seen in Fig. 5b, all samples had the same two peaks around 284.6 Ev and 532 Ev. The first peak (around 284.6 Ev) was related to C1s core level and the second peak (around 532 Ev) was related to O1s^61^. The intensity peak O1s was weak in pure SWCNT and after being functionalized with DSPE-PEG-COOH, its value increased. On the other hand, the oxygen to carbon (O/C) ratio increased from 0.023 for pure SWCNT to 0.274 for FSWCNT. These results confirmed the successful non-covalent functionalization of SWCNT, and the existence of DSPE-PEG-COOH on the side-wall surface of SWCNT. Furthermore, this ratio did not change significantly for DFSWCNT relative to the one for FSWCNT. However, O/C ratio for DFSWCNT-Amb increased to 0.452, indicating the existence of oxygen atoms in Amb structure. Finally, it could be concluded that the functionality of SWCNT and Amb loading was a successful process.

**Table 1:** Results of Raman analysis.

Name	D-band		G-band		Gʹ-band		I_G_/I_D_
	Raman shift (cm^−1^)	Intensity	Raman shift (cm^−1^)	Intensity	Raman shift (cm^−1^)	Intensity	
Pure SWCNT	1341	5465.1	1585	39,076.6	2674	12,613.4	7.15
FSWCNT	1347	21,048.8	1588	86,060.8	2682	33,560.3	4.08
DFSWCNT	1345	35,075.6	1588	137,496	2679	65,926.6	3.91

### SEM and TEM

**Figure 6:**
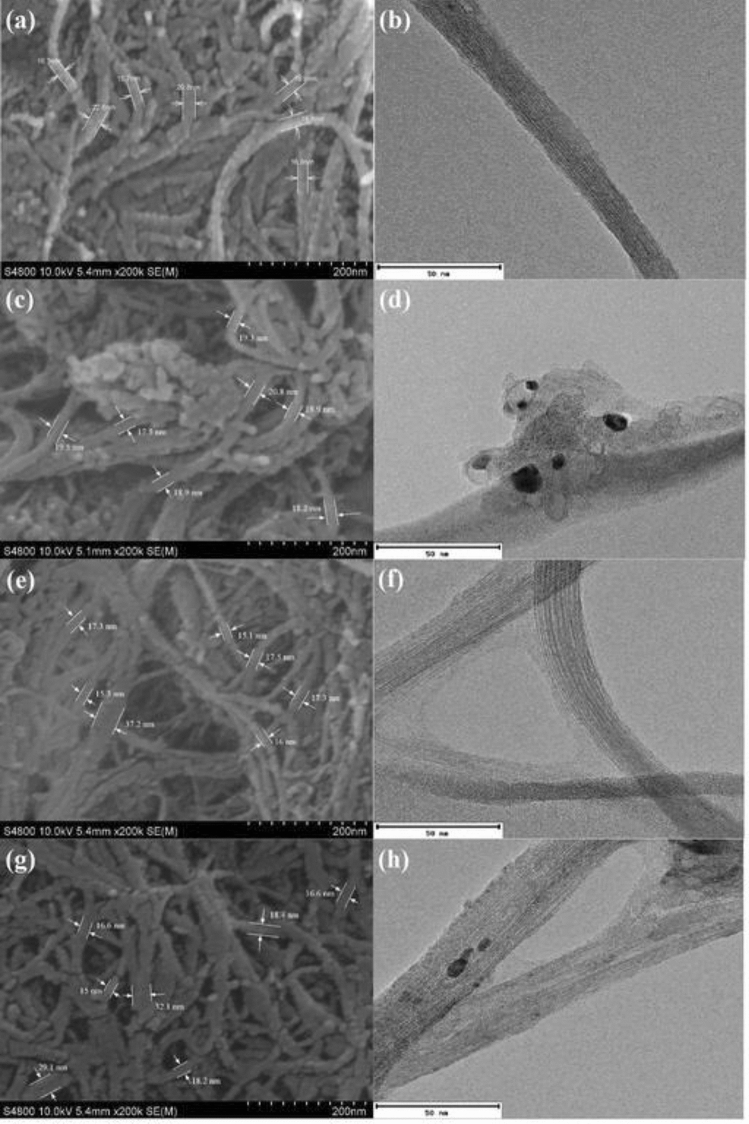
SEM and TEM images of (**a**,**b**) pure SWCNT, (**c**,**d**) FSWCNT, (**e**,**f**) DFSWCNT and (**g**,**h**) DFSWCNT-Amb, respectively.

SEM and TEM analyses were carried out to investigate the morphology of SWCNT’s surface before and after functionalization, and also when it was applied as the Amb nanocarrier. Figure 6 displays the SEM and TEM images of pure SWCNT, FSWCNT, DFSWCNT and DFSWCNT-Amb. The SEM and TEM images of pure SWCNT displayed networks of smooth and uniform tubes with diameters less than 100 nm. After measuring the diameter of SWCNT in SEM images, it was observed that the average diameters of SWCNT before and after being functionalized with DSPE-PEG-COOH were 18.42 ± 0.05 nm and 19.02 ± 0.07 nm, respectively. It means that the functionalized SWCNT’s diameter increased relative to pure SWCNT and a thin layer of DSPE-PEG-COOH formed a harsh surface on the side wall surface of SWCNT. In addition, the dispersion of SWCNT improved after functionalization and fewer aggregations were visible. Besides, the length of SWCNT decreased, by fracturing into shorter SWCNTs. Also, it can be seen in Fig. 6e–h that the average diameters of DFSWCNT and DFSWCNT-Amb were 19.38 ± 0.04 nm and 20.86 ± 0.05 nm, respectively. It can be concluded that the attachment of EDA to FSWCNT had a small effect on the diameter of FSWCNT. In other words, thin layers of EDA and Amb coated the side wall surface of FSWCNT, and concealed the surface of DFSWCNT. Overall, each conjugation to the side wall surface of SWCNT was visible as a thin layer in SEM and TEM images. Hence, SWCNT had a maximum diameter of 20.86 ± 0.05 nm when Amb was loaded on it. These morphology results of functionalized CNT were in agreement with other researches that have shown a thin layer of polymer emerges on the surface of CNT after modification and CNT’s diameter increases. Also CNT’s solubility improved and its agglomeration intensity reduced^73,74^. The magnifications of SEM and TEM images were x200k and x20k, respectively.

### Amb’s calibration curve

Six samples with different pure Amb concentrations were made by diluting Amb with phosphate buffered saline (PBS). The absorbance of each sample was read by UV–visible spectrophotometer at maximum peak (410 nm). Supplementary Figure S4 displays the calibration curve of Amb at 410 nm and at neutral (7.4) and acidic (5.5) pHs. A linear curve was obtained by linear regression analysis of the corresponding plot points, and used for calculating the amount of Amb released in PBS medium when passing through the dialysis bag.

### In vitro Amb release study

**Figure 7:**
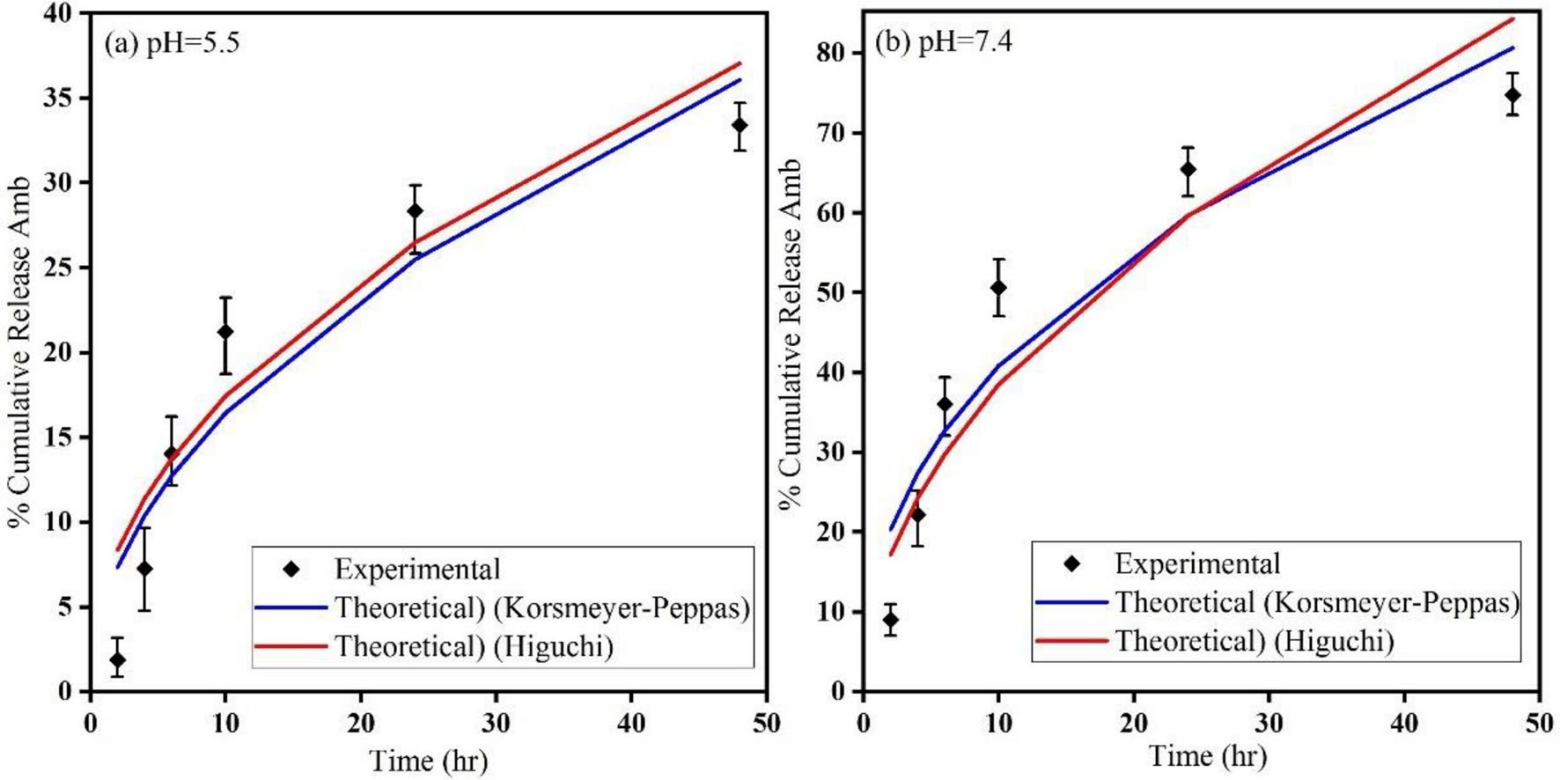
Amb Cumulative release profile from DFSWCNT-Amb formulation in PBS medium. Higuchi and Korsmeyer-Peppas kinetic models of Amb release from DFSWCNT at (**a**) pH = 5.5 and (**b**) pH = 7.4.

Drug release profiles versus time at two pHs were compared with each other as shown in Fig. 7. It can be observed that about 33% and 75% of Amb with corresponding pHs of 5.5 and 7.4 were released after 48 h. The percentage of cumulative release values show that Amb had a persistent and sustained release rate in neutral pH (7.4) compared to the acidic medium (pH = 5.5). Therefore, it can be concluded that the pH of release medium played a significant role in the quantity released and the kinetics of Amb from DFSWCNT formulation. At pH = 7.4, the interaction between Amb and release medium was stronger than the interaction between Amb and DFSWCNT, and because of this, Amb would likely detach from nanocarrier and enter the release medium. In other words, the amide bond between the Amb and EDA broke easily in the high pH medium. Conversely, in acidic medium (pH = 5.5), the interaction between DFSWCNT and Amb pair was more powerful than that of Amb and release medium pair, and Amb’s releasing was slow. Finally, some parameters such as interactions between components (DFSWCNT, Amb and release medium) and Amb solubility in the release medium effected Amb’s release process. This kind of Amb release study has been reported before for other polymeric nanoparticles acting as nanocarriers^58,75,76^.

Many different kinetic models (zero-order, first-order, Higuchi and Korsmeyer-Peppas models^77–79^ should be applied to describe Amb’s release and mass transport mechanisms. Accordingly, the in vitro scale Amb release data of DFSWCNT-Amb nanocarrier obtained at the two above mentioned pHs should be fitted into various kinetic models. Zero-order model is one of the drug release kinetic models with a constant drug release rate over a period of time. However, it only depends on time. The first-order model is another drug kinetic release model, where the drug release rate is dependent on the concentration of the drug in the release medium. The next model is a Higuchi model based on Fickian diffusion law, that describes the release rate as a function of the square root of time^80^. Furthermore, there is another model called Korsmeyer-Peppas model, which is the generalized form of Higuchi model. The equations describing all the above mentioned models are displayed in Table 2, where, ${M}_{t}$ is the absolute cumulative amount of drug released at time t, ${M}_{\infty }$ is the absolute cumulative amount of drug released after infinite time, k is the release kinetic constant and n is the diffusion exponent parameter.

**Table 2:** Equations of kinetic release models and their corresponding evaluated R^2^ values in vitro scale release study with two different pHs.

Name of model	Equation	R^2^ at pH = 5.5	R^2^ at pH = 7.4
Zero-Order	${\mathrm{M}}_{\mathrm{t}}={\mathrm{M}}_{\infty }+\mathrm{kt}$	0.880	0.868
First-Order	$\mathrm{ln}\left(\frac{{\mathrm{M}}_{\mathrm{t}}}{{\mathrm{M}}_{\infty }}\right)=\mathrm{kt}$	0.70	0.730
Higuchi	$\frac{{\mathrm{M}}_{\mathrm{t}}}{{\mathrm{M}}_{\infty }}=\mathrm{k}\sqrt{\mathrm{t}}$	0.947	0.940
Korsmeyer-Peppas	$\mathrm{ln}\left(\frac{{\mathrm{M}}_{\mathrm{t}}}{{\mathrm{M}}_{\infty }}\right)=\mathrm{lnk}+\mathrm{nlnt}$	0.947 (n = 0.5)	0.948 (n = 0.433)

The range of R^2^ value is between zero and 1, and it indicates the quality of the regression. If this value is equal to 1, it means that the experimental data has an excellent agreement with the theoretical data. As the results of R^2^ values for various release kinetic models in Table 2 show, the zero-order and first-order models had non-satisfactory R^2^ values and far from unity. However, in the case of Higuchi and Korsmeyer-Peppas models, the R^2^ values were closer to unity and described the existence of good agreement between experimental and theoretical sets of data. The choice of proper values for diffusion exponent parameter (n) in Korsmeyer-Peppas model was an important parameter decision for achieving excellent fit of the experimental data. The values of (n) are 0.5 and 0.433 at pHs 5.5 and 7.4, respectively. Furthermore, the value of n gave a significant piece of information about the drug release mechanism. When the value of n was lower than 0.45 (0.433 with pH = 7.4), the release mechanism was quasi Fickian and when it was between o.45 and 0.89 (0.5 with pH = 5.5), the release mechanism was non-Fickian diffusion or anomalous diffusion^78,81^. Note that the value of n at pH = 5.5 was 0.5 and in this case, both Higuchi and Korsmeyer-Peppas models acted similarly, and had the same R^2^ value and a good fit of the experimental data. Both Higuchi and Korsmeyer-Peppas kinetic models with a maximum R^2^ values at two pHs (5.5 and 7.4) are presented in Fig. 7.

### Cytotoxicity studies

#### Cytotoxicity assay of CNTs before and after functionalization

**Figure 8:**
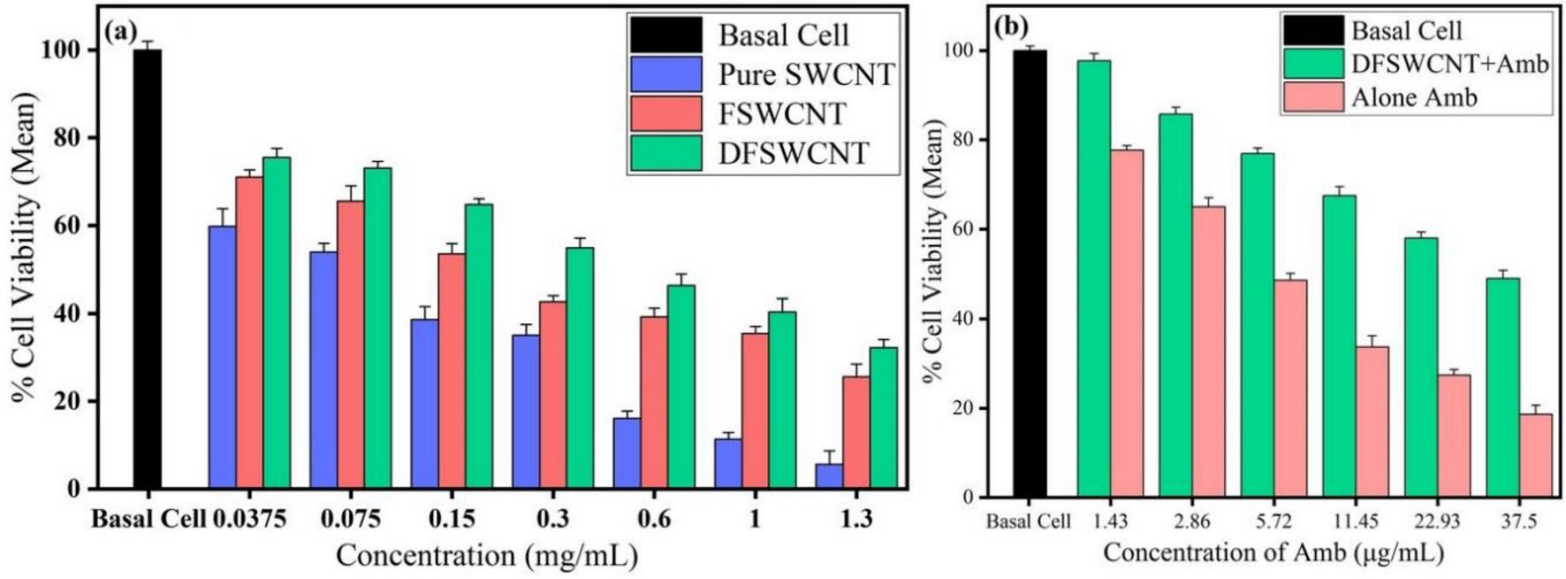
Biocompatibility assays in HEK-293 cells. (**a**) Biocompatibility of carbon nanotubes at different stages of functionalization and at concentrations ranging from 0.0375 to 1.3 mg/mL, (**b**) biocompatibility of Amb and DFSWCNT-Amb at drug concentrations ranging from 1.43 to 37.5 µg/mL.

The cells exposed at different concentrations (0.075–1 mg/mL) of pure SWCNT, FSWCNT and DFSWCNT, showed an increase in toxicity with increasing concentrations of carbon nanotubes. As can be observed in Fig. 8a, the pristine SWCNT had higher toxicity than both FSWCNT and DFSWCNT at all concentrations. So, we could conclude that functionalization of SWCNT helped to decrease its high toxicity level. In addition, cytotoxicity results showed that polymer DSPE-PEG-COOH polymer was a good candidate to functionalize SWCNT, not only because it was a biocompatible derivative of PEG polymer, but also because its toxicity effect would decrease due to its capacity to improve dispersability and aqueous solubility^82–84^. These results are in agreement with other researches^84,85^ that have confirmed the considerable effect of CNT functionalization on decreasing the toxicity of pure CNT. The cell viability percentage of FSWCNT was 35.45%, which is higher than pure SWCNT (11.40%) at 1 mg/mL concentration. Also, the cell viability percentage of DFSWCNT nanocarrier with 0.6 mg/mL concentration was 46.30%. This value is higher than cell viability percentage values of pure SWCNT (16.14%) and FSWCNT (39.25%) at the same concentration. Finally, we could conclude that functionalization of pure SWCNT with biocompatible ingredients decreased the toxicity level of pure SWCNT and may trigger the possibility of using these nanocarriers as suitable vectors for drug and gene delivery.

#### Cytotoxicity analyses of both free and loaded Amb on DFSWCNT

As the results show in Fig. 8b, the cytotoxicity of pure Amb on HEK-293 cells was higher than the cytotoxicity of Amb loaded on DFSWCNT at all concentration values. In our Amb delivery system, the concentration of DFSWCNT-Amb was 22.93 µg/mL and the percentages of cell viability of DFSWCNT-Amb and Amb were 58% and 27.35%, respectively. So we could conclude that the delivery of Amb loaded on the surface of DFSWCNT caused less toxicity than pure Amb. These results are consistent with other studies conducted on the toxicity of Amb on HEK-293 cells^86,87^. In other words, to decrease the toxicity of Amb and improve Amb delivery, researchers have attempted to reduce this undesirable feature by designing delivery systems that involve attaching long chain fatty acids to Amb^88,89^, developing lipid systems for Amb delivery^87^ and evaluating toxicity of Amb on HEK-293 cells^86,90^.

#### Gene delivery efficiency

**Figure 9:**
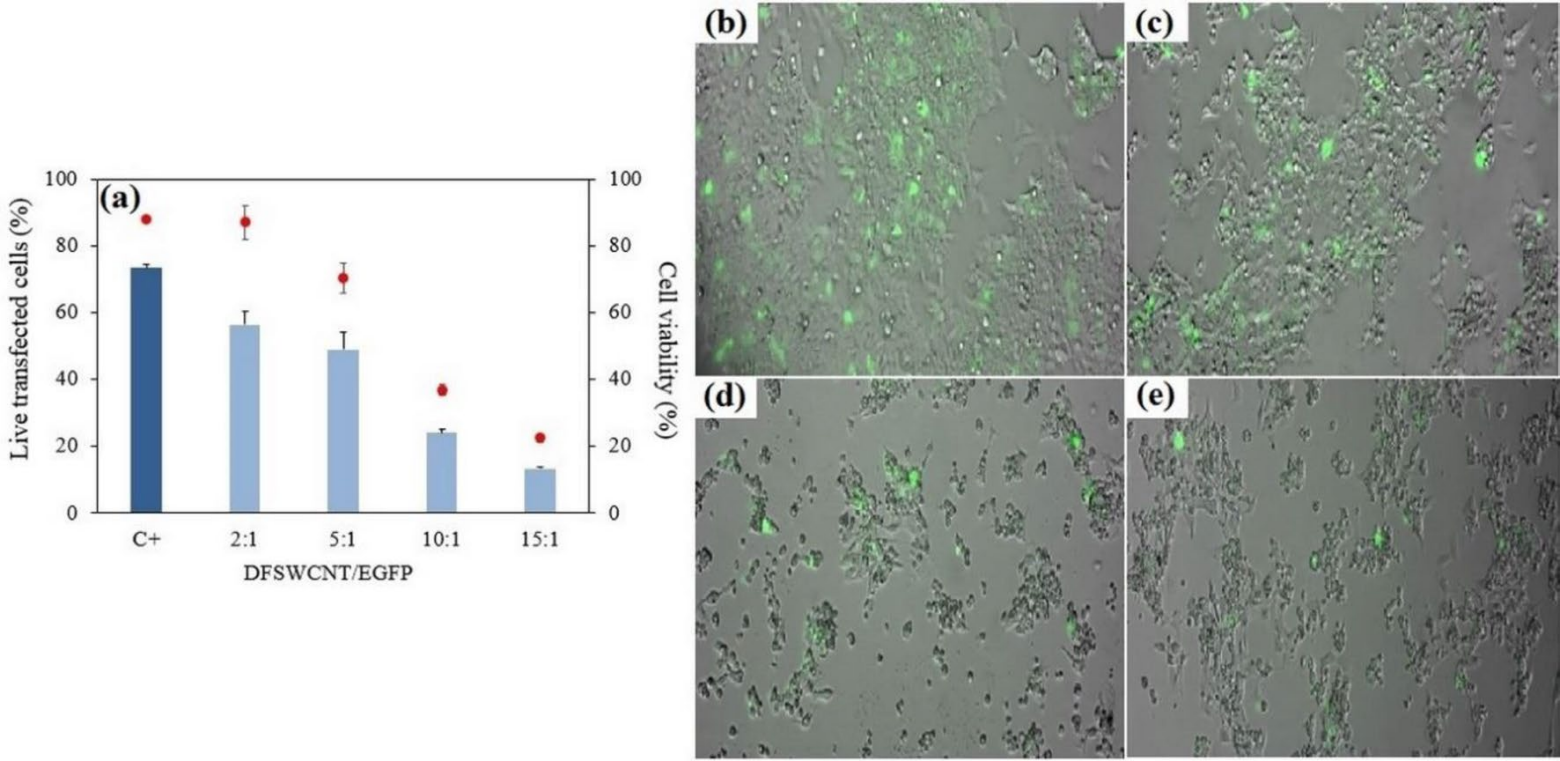
Transfection efficiency of DFSWCNT. (**a**) Percentage of EGFP positive live cells (bar) and cell viability (dots) of DFSWCNT vectoring EGFP plasmid at different DFSWCNT/EGFP plasmid mass ratios in HEK-293 cells evaluated by flow cytometry. Data are presented as mean ± SD of three measurements. Fluorescence microscope images of EGFP signal in HEK-293 cells transfected with DFSWCNT complexed with EGFP plasmid at (**b**) 2:1, (**c**) 5:1, (**d**) 10:1 and (**e**) 15:1 DFSWCNT/pEGFP mass ratios.

Transfection assays were performed using DFSWCNT (as a vector of EGFP) at DFSWCNT/EGFP plasmid mass ratios of 2:1, 5:1, 10:1 and 15:1 in HEK-293 cells. The results are shown in Fig. 9a. It can be observed that the maximum percentage of cell viability and live transfected cells correspond to the complex with 2:1 ratio. After increasing the DFSWCNT/EGFP plasmid mass ratio, the percentage of the cell viability and EGFP expression cells started to decrease, and a considerable decrease happened when the ratio changed from 5:1 to 10:1. In other words, the cell viability for 10:1 ratio (36.66 ± 1.56%) was about half of the value for 5:1 ratio (70.22 ± 4.52%). Similarly, the transfection efficiency of the 10:1 ratio (23.96 ± 1.20%) was almost half of the transfection efficiency of 5:1 ratio (48.95 ± 5.22%). The transfection with positive control lipofectamine™ 2000 showed 73.27 ± 1.06% of EGFP expressing live cells and a cellular viability value of 88.09 ± 0.24%. Transfection results showed that DFSWCNT can be applied as a vector for EGFP plasmid at DFSWCNT/EGFP plasmid mass ratio 2:1 or 5:1 which had high live transfected cells as well as high cell viability percentage. So, DFSWCNT could act as a vehicle in gene delivery systems^91–93^ such as cationic niosomes^94–96^. The results of qualitative analysis of transfection efficiency via examination of HEK-293 cells under fluorescence microscopy 48 h after transfection can be observed in Fig. 9b–e.

## Conclusions

In this study, pure SWCNT was initially functionalized non-covalently with a phospholipid carboxilic acid derivative of PEG (DSPE-PEG(5000)-COOH) through π-π stacking between the hydrophobic surface of prisitine SWCNT and hydrophobic chain of DSPE-PEG-COOH. Then, the PEGylated SWCNT was conjugated to EDA by formation of amid bonds between the carboxilic acid groups of DSPE-PEG-COOH and the amine groups of EDA. Characterization results confirmed the successful conjugation of DSPE-PEG-COOH to SWCNT and EDA to PEGylated SWCNT. Also, the results indicated that dispersity, solubility and cytotoxicity of SWCNT improved after functionalization. The modified SWCNT was applied as a vehicle for Amb and EGFP plasmid delivery. The release of Amb was investigated via UV–visible analysis, which showed that it had a sustained release in neutral medium as opposed to in acidic medium. The Higuchi and Korsmeyer-Peppas kinetic models with R^2^ values more than 0.9 were chosen as the best mode for experimental sets of data. In addition, the cytotoxicity of functionalized SWCNTs (PEGylated SWCNT and DFSWCNT) were lower than that of pure SWCNT against HEK-293 cells. It can be concluded that functionalizing of SWCNT with biocompatible ingredients played a significant role in reducing cytotoxicity of pure SWCNT and in this way, SWCNT could be applied in therapeutic scenarios. Also, the cytotoxicity of Amb loaded on modified SWCNT formulation was lower than the cytotoxicity of pure Amb, which shows that this formulation is an appropriate system for Amb delivery. Furthermore, this modified SWCNT was used for EGFP plasmid delivery and the results indicated that CNTs have the capability to be novel non-viral vectors for gene delivery applications in gene delivery systems.

## Supplementary Information

Supplementary Information.

## Data Availability

The authors declare that the data supporting the findings of this study are available within the article and its supplementary information file.
