# Supplementary material for: Design of double functionalized carbon nanotube for amphotericin B and genetic material delivery
Source: Sci Rep. 2022 Dec 7;12:21114. doi: 10.1038/s41598-022-25222-1 (PMC9729229; doi:10.1038/s41598-022-25222-1)
Supplement: Supplementary file 1 — Supplementary Information. [file 41598_2022_25222_MOESM1_ESM.docx]

Supplementary Material:

Design of Double Functionalized Carbon Nanotube for Amphotericin B and Genetic Material Delivery

Sara Yazdani, Mehrdad Mozaffarian*, Gholamreza Pazuki, Naghmeh Hadidi, Idoia Gallego, Gustavo Puras and Jose Luis Pedraz*

**Table S1.** Thermogravimetric (TGA) results of pure SWCNT, FSWCNT, DFSWCNT and DFSWCNT-Amb.

| **Name** | **%Total mass loss** | **%Mass loss related to impurities of SWCNT** | **%Mass loss related to DSPE-PEG-COOH** | **%Mass loss related to EDA** | **%Mass loss related to Amb** |
| --- | --- | --- | --- | --- | --- |
| **Pure SWCNT** | 6.5920% | 6. 92% | - | - | - |
| **FSWCNT** | 93.04% | 6. 92% | 86.15% | - | - |
| **DFSWCNT** | 95.10% | 6. 92% | 66.54% | 21.50% | - |
| **DFSWCNT-Amb** | 97.09% | 6. 92% | 58% | 20.09% | 12.21% |

**Table S2.** Results obtained from C1s high resolution spectra

|  | **Name** | **BE** | **%Concentration** | **% Carbon** |
| --- | --- | --- | --- | --- |
| Pure SWCNT | C-C (Sp^2^), C-H  C-C (Sp^3^)  C-O  O-C=O | 284.6  285.2  286.4  288.4 | 54.1  14.3  11.4  4.0 | 97.2 |
| FSWCNT | C-C (Sp^2^), C-H  C-O  O-C=O | 284.6  286.1  288.4 | 39.3  33.6  3.5 | 76.4 |
| DFSWCNT | C-C (Sp^2^), C-H  C-N, C-O  N-C=O, O-C=O | 284.6  286.2  288.1 | 16.7  48.6  4.5 | 69.9 |
| DFSWCNT-Amb | C-C (Sp^2^), C-H  C-N, C-O  N-C=O, O-C=O | 284.6  286.1  287.7 | 7.8  53.5  2.5 | 63.9 |

**Table S3.** Results obtained from O1s high resolution spectra

|  | **Name** | **BE** | **%Concentration** | **% Oxygen** |
| --- | --- | --- | --- | --- |
| Pure SWCNT | -COO, C=O | 531 | 1.3 | 2.3 |
|  | C-OH | 533 | 1.0 |  |
| FSWCNT | -COO, C=O | 531 | 2.9 | 21 |
|  | C-OH | 532.5 | 18.1 |  |
| DFSWCNT | -COO, C=O | 530.6 | 4 | 22.5 |
|  | C-OH | 532.4 | 18.5 |  |
| DFSWCNT-Amb | -COO, C=O | 531 | 3.6 | 28.9 |
|  | C-OH | 532.3 | 24.3 |  |
|  | H_2_O | 535.4 | 0.95 |  |

**Table S4.** Results obtained from N1s high resolution spectra

|  | **Name** | **BE** | **%Concentration** | **% Nitrogen** |
| --- | --- | --- | --- | --- |
| FSWCNT | N1s | 400 | 1.11 | 1.11 |
| DFSWCNT | N1s | 399.2 | 3.78 | 5.21 |
|  | N1s | 401.0 | 1.43 |  |
| DFSWCNT-Amb | N1s | 399.1 | 3.62 | 5.50 |
|  | N1s | 401.0 | 2.88 |  |

**Table S5.** Results obtained from P2p high resolution spectra

|  | **Name** | **BE** | **%Concentration** | **% Phosphorus** |
| --- | --- | --- | --- | --- |
| FSWCNT | P2p | 133.7 | 0.31 | 0.31 |
| DFSWCNT | P2p | 134.3 | 0.32 | 0.32 |
| DFSWCNT-Amb | P2p | 133.7 | 0.31 | 0.31 |

**Table S6.** wide-scan C1s and O1s XPS spectra results

| **Name** | **C1s** | | **O1s** | |
| --- | --- | --- | --- | --- |
|  | **Binding Energy (eV)** | **Intensity** | **Binding Energy (eV)** | **Intensity** |
| Pure SWCNT | 284.592 | 144398 | 532.592 | 20991.1 |
| FSWCNT | 284.602 | 73229.6 | 531.602 | 71934.9 |
| DFSWCNT | 286.192 | 304570 | 532.192 | 304515 |
| DFSWCNT-Amb | 286.098 | 297894 | 532.098 | 372352 |


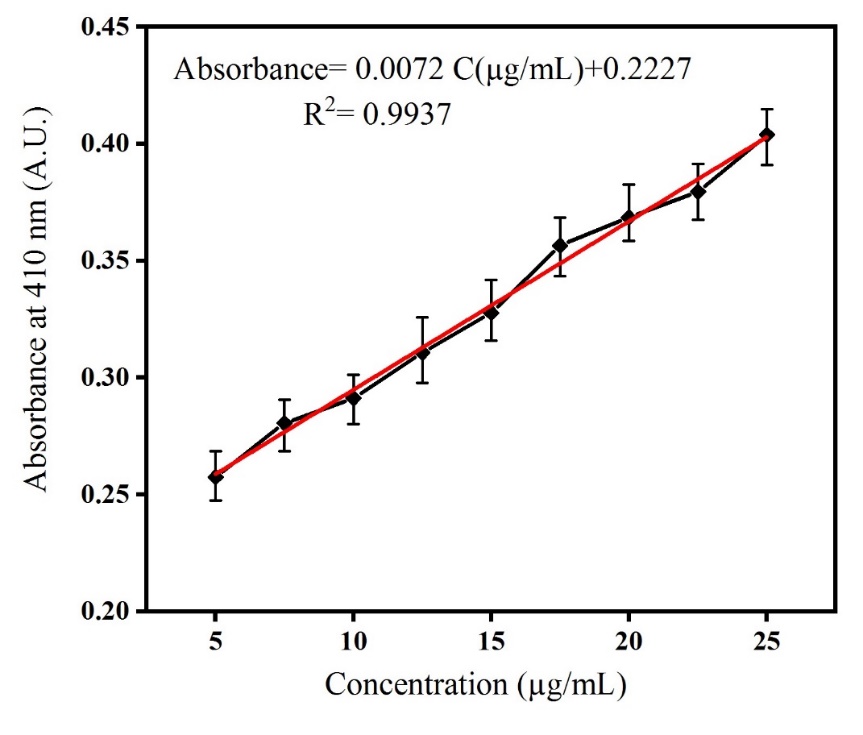


**Figure S1.** Calibration curve of Amb in Milli-Q water at 410 nm wavelength.


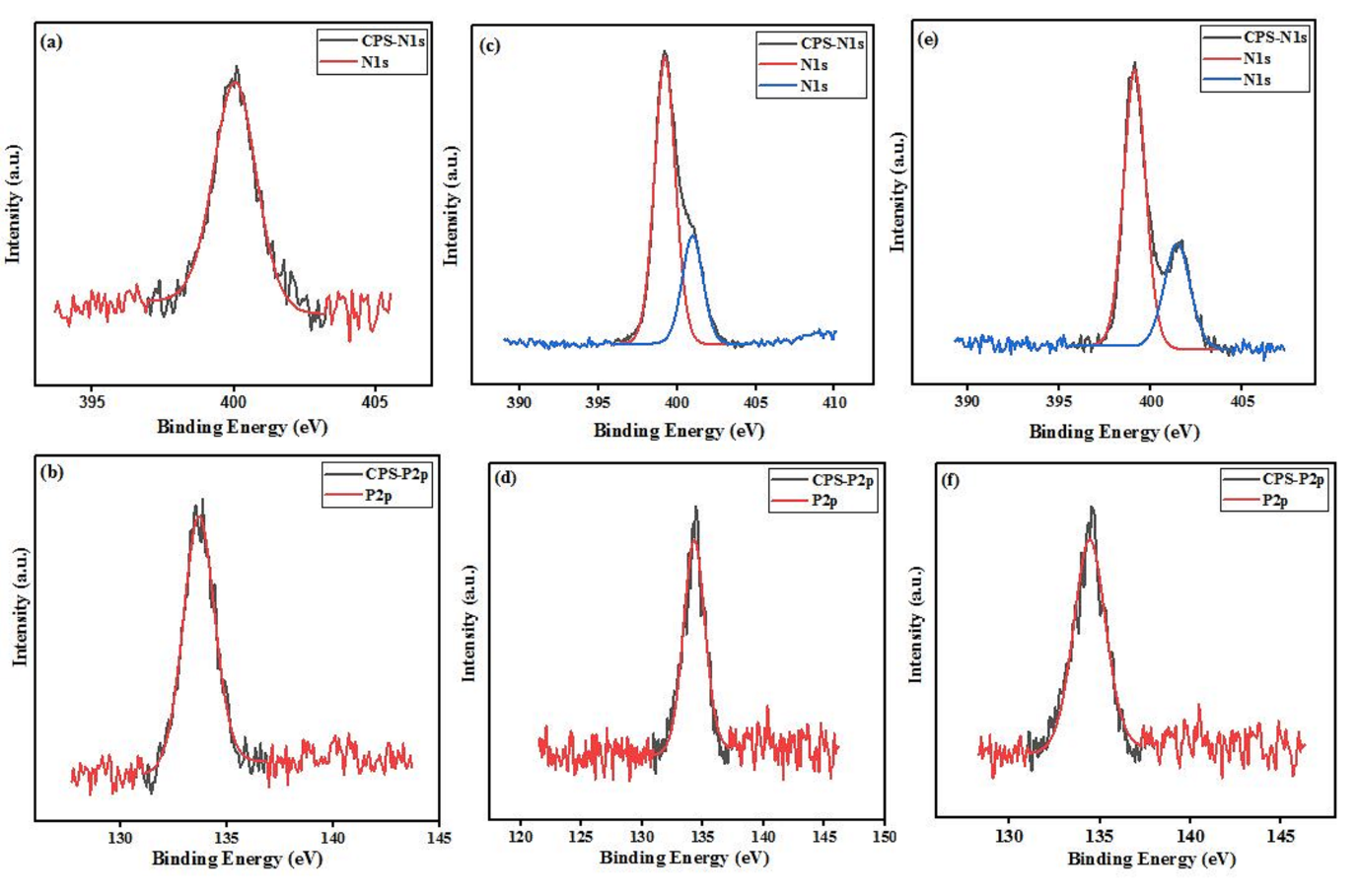


**Figure S2.** X-ray photoelectron spectroscopy (XPS) N1s and P2p high resolution spectra (a) N1s FSWCNT, (b) P2p FSWCNT, (c) N1s DFSWCNT, (d) P2p DFSWCNT, (e) N1s DFSWCNT-Amb and (f) P2p DFSWCNT-Amb.


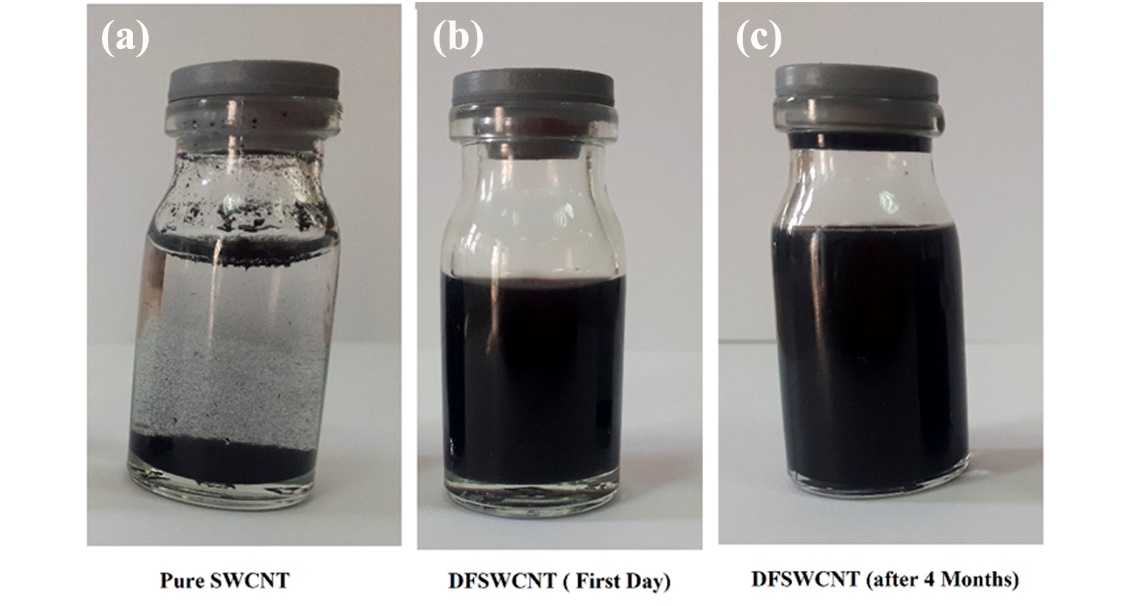


**Figure S3.** Digital photos of pure SWCNT and DFSWCNT dispersion at different time


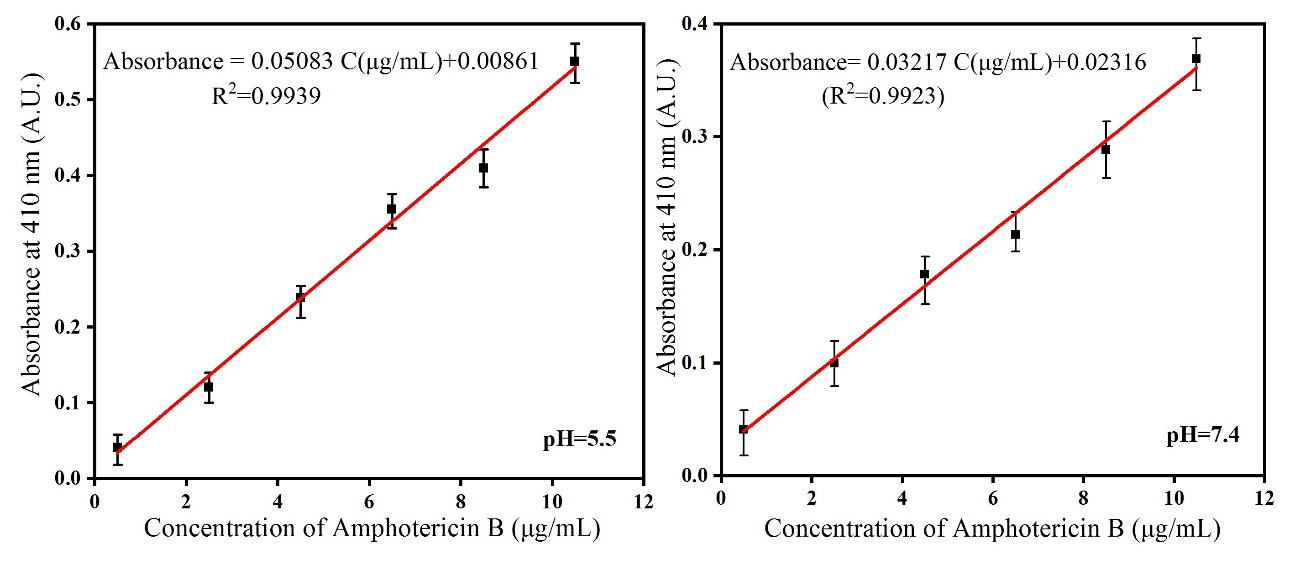


**Figure S4.** Calibration Curves of Amb at 410 nm (pH=5.5 and pH=7.4).
